# Oöphorectomy in Cancer

**Published:** 1902-01-11

**Authors:** 


### OÖPHORECTOMY IN CANCER.

In a clinical lecture recently delivered at St. Bartholomew's Hospital Mr. Butlin [1] very carefully reviewed the present position of oöphorectomy in the treatment of cancer of the breast. Undoubtedly some of the cases which have been recorded are very surprising, so far as the immediate results of the operation are concerned, and at the least they tend to show that, however local the cancer process may be in its origin, it is to a large extent under the control of general condition, and that some such disturbance of nutrition as occurs in the breasts after removal of the ovaries and as is produced throughout the body by the administration of thyroid extract may in certain cases, at any rate for a time, not only put a stop to cancer growth, but cause the retrogression of cancerous masses which have already developed. When we come, however, to estimate the position which ought to be taken by oöphorectomy in the treatment of the disease it is extremely important that we should not be led astray by curious and abnormal cases. This warning would hold good even if now and again the successes we hear of were permanent. But, according to what Mr. Butlin now tells us, permanence is just what these apparent cures lack. He refers to the after-history of several of the cases of which so much has been made, and shows that, brilliant as the results appeared at the time, the disease in the end returned; hence his verdict is far from favourable. Indeed, in cases in which removal of the growth by local operation is possible, he is absolutely opposed to wasting time by attempting to modify its progress by oöphorectomy or any such measures. In order to place the issue clearly, he points out that the advantages of the local operation in cancer of the breast are not exclusively restricted to those cases in which cure results. He describes three groups of cases:—(1) Take a woman who is suffering from a small cancer of the breast, with no affection of the glands, and there is a reasonable hope that, by a considerable operation, one may cure that patient in so far as she will not have cancer again. She is just as much cured as a patient is who has had pneumonia. She may remain 10, 15, or 20 years without any cancer, and may die of some other disease. (2) Again, take a woman who has cancer of the breast with enlarged axillary glands. These are taken away by a sufficient operation. The disease recurs *in situ*. That means failure of the operation up to a certain point. But, after all, she may be much less miserable, and may die a much less painful death than if the operation had never been performed. (3) Then there are certain cases in which no recurrence takes place *in situ*, but the disease crops up again in some other part of the body. But there is no ulcerated mass, and the patient dies in much less misery. Thus we may hold out three forms and degrees of reward for undergoing the operation: first, the hope, which we can now hold out to a goodly number of women, that the operation may be quite successful; secondly, that if the disease kills, it may do so by occurring in some distant organ, and with much less pain and suffering; thirdly, that, even if it recurs *in situ* in the form of nodules, the patient will suffer far less than if no operation had been performed. That being so, Mr. Butlin asks what can be said for oöphorectomy and the administration of thyroid extract? (1) So far as is known there is not a single case of cure, such cure as one gets by local operation; (2) when the disease comes back it does so in exactly the same form and with all the same trouble as before, and the manner and distress of the patient's death are not in the least changed by the procedure; and (3) it has to be admitted that a very large number of women on whom oöphorectomy has been performed and who have been soaked in thyroid extract have not received the smallest benefit in the world. Of course it has to be remembered that the oöphorectomy and thyroid treatment have for the most part only been employed in very bad cases, but as the matter stands at present Mr. Butlin's remarks go strongly to emphasise the line we always advocate on this subject, viz., that local operations should be done, and done early. Think for a moment of what Sir William Banks has said on the subject: "Have you ever imagined," he says, "what the results would be if all cancers were thoroughly excised when they were no bigger than peas?" Let us then have no temporising, no watching of the case, no waiting for developments.

[1] Brit. Med. Jour., Jan. 4.

